# A mathematical model for designing networks of C-Reactive Protein point of care testing

**DOI:** 10.1371/journal.pone.0222676

**Published:** 2019-09-17

**Authors:** Carlos Lamas-Fernandez, Gail Hayward, Michael Moore, Thomas Monks

**Affiliations:** 1 NIHR CLAHRC Wessex, University of Southampton, Southampton, United Kingdom; 2 NIHR Community Healthcare MIC, Nuffield Department of Primary Care Health Sciences, University of Oxford, Oxford, United Kingdom; 3 Primary Care and Population Sciences, University of Southampton, Southampton, United Kingdom; 4 University of Exeter Medical School, University of Exeter, Exeter, United Kingdom; Cambridge University, UNITED KINGDOM

## Abstract

One approach to improving antibiotic stewardship in primary care may be to support all General Practitioners (GPs) to have access to point of care C-Reactive Protein tests to guide their prescribing decisions in patients presenting with symptoms of lower respiratory tract infection. However, to date there has been no work to understand how clinical commissioning groups might approach the practicalities of system-wide implementation. We aimed to develop an accessible service delivery modelling tool that, based on open data, could generate a layout of the geographical distribution of point of care facilities that minimised the cost and travel distance for patients across a given region. We considered different implementation models where point of care tests were placed at either GP surgeries, pharmacies or both. We analysed the trade-offs between cost and travel found by running the model under different configurations and analysing the model results in four regions of England (two urban, two rural). Our model suggests that even under assumptions of short travel distances for patients (e.g. under 500m), it is possible to achieve a meaningful reduction in the number of necessary point of care testing facilities to serve a region by referring some patients to be tested at nearby GP surgeries or pharmacies. In our test cases pharmacy-led implementation models resulted in some patients having to travel long distances to obtain a test, beyond the desired travel limits. These results indicate that an efficient implementation strategy for point of care tests over a geographic region, potentially building on primary care networks, might lead to significant cost reduction in equipment and associated personnel training, maintenance and quality control costs; as well as achieving fair access to testing facilities.

## Introduction

The measurement of C-Reactive Protein (CRP) at the point of care (PoC) in patients presenting symptoms of lower respiratory tract infections (LRTI) can support appropriate antibiotic prescribing in primary care. A recent guideline by the National Institute for Clinical Excellence (NICE) on pneumonia provided guidelines on prescribing based on concentration boundaries [1].

There is evidence [2–5] that CRP tests can reduce the antibiotic prescribing rates for LRTI, without affecting patient’s recovery. However, despite the evidence and availability of guidelines, the uptake of these techniques in England seems to be limited [6,7], especially in comparison to other countries [8]. A number of barriers to widespread implementation have been identified (such as changes to General Practitioner’s (GP) workflow or operational constraints), but a key barrier is the cost of machine and cartridge purchases and maintenance. The cost of one of the testing machines considered in this study and its quality assurance costs are £1200 and £800 per year, respectively. Quality control costs can vary depending on implementations, but can be as much as £470 per machine per year for stand-alone systems (18). If clinical commissioning groups (CCGs) were to become responsible for the financial support required for a system wide roll out of PoC CRP testing, it would be important to use an approach which minimised the number of machines required, and therefore the associated costs, without limiting patient access to testing.

In this study we aimed to provide commissioners with practical guidance on how a CRP PoC testing network could be implemented if a decision was made to fund it. We compared testing patients in their own GP surgeries to redirecting them to be tested at other nearby locations (pharmacies or other GP surgeries). GPs (family doctors) are the first point of contact with the health service for the majority of patients and, therefore, GP surgeries are well distributed geographically across the country. In these shared-resource models, suspected LRTI patients would be referred to these facilities by their usual GPs following clinical assessment, if a CRP test was considered necessary. In a pharmacy model, patients would be issued an antibiotic prescription by their GP which would only be ‘cashed’ if the test result was above a required level. The choice between these models has an impact on both commissioner resources (the need to acquire more or less testing devices) and the patients (who might need travel longer or shorter distances to be tested).

## Aims

This study is set out to develop an automated optimisation tool that provides commissioners with optimal tactical planning decisions (e.g. allocation of patients to testing locations) based on a number of inputs. These inputs are based on the commissioner’s strategic decisions, for example, whether to allow pharmacies to perform tests or not; or what is the maximum acceptable travel distance for a patient. The main outcome measures of the model are the number of machines required and the expected extra travel distances for patients to be tested. The results of the model were analysed for different locations (urban and rural) and for various combinations of strategic decisions.

## Methods

When investigating alternative locations for PoC testing a key decision that determines the costs and travel implications of the service delivery implementation is the quantity and physical location of PoC facilities. To determine optimal values for them under the different implementation models investigated, we developed a mathematical location-allocation model that considers two decisions. The first one is to determine the minimum number of testing machines needed to cover the population of the considered geographical region. The second one is to determine their location and which patients should be assigned to them, in order to minimise the extra travel patients might have to perform. To avoid excessive travel distances for some patients the maximum allowed travel per patient is subject to an upper limit.

In the remainder of this section, we describe the data used for the model, the model itself, and the different configurations we used to test the model.

### Data sources

In order to determine the layout of a CRP PoC deployment, our model requires the following information:

- Geographical locations of GPs and pharmacies which could potentially host a CRP PoC testing facility
- Testing capacity of a single machine, in number of tests per week
- Demand for tests in a census Output Area (OA), in number of tests per person and per week
- Estimations of the extra distance travelled by patients if they were receiving a test in each of the potential testing facilities

The locations of GP surgeries (including their branches) and pharmacies are open data that can be accessed in [9] and [10] respectively.

The testing capacity of a machine was estimated at 175 tests per week, using as a reference the capacity of GPs for consultations. Given the uncertainty in the incidence of LRTI and the clinician’s approach to testing, we calculated low and high estimates of demands of tests per person and per week. We provide an analysis of the effect of using each of these estimates.

In order to take into account the geographical distribution of the population within the considered geographical regions, we have used census Output Areas. OAs are the lowest geographical level at which the UK census provides information and are constructed with the idea of preserving homogeneity within their population.

We refer the reader to the online appendix **S1 Appendix** for further details on how these demand and capacity estimates were calculated, as well as information on the geographical regions considered and how the GP location data can be obtained.

**Fig 1:**
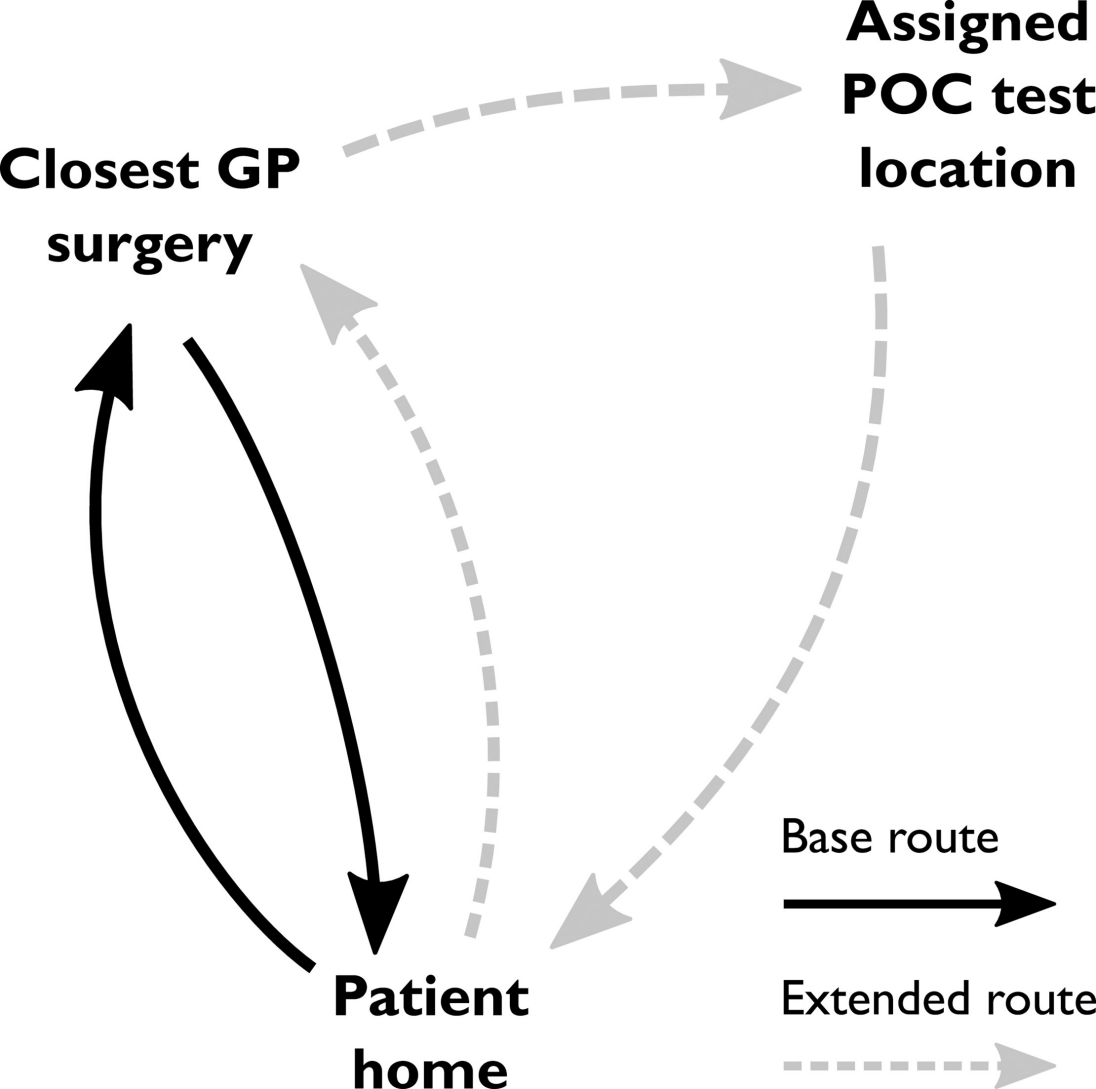
Schematic of the base and extended travel routes for a patient tested elsewhere than their closest GP. The travel burden is calculated as the difference of the two.

A central part of our modelling approach is estimating the travel burden, that is, the extra distance that patients would need to travel to be tested in a location other than their usual GP surgery. To compute this distance, we first calculated the base travel that patients would always perform, even if not getting a test: the distance of a return trip from their home to their closest GP surgery. Since this distance was not explicitly available, we approximated it as the distance from the centroid of their origin OA to the location of the closest GP surgery. When the patients are assigned to be tested somewhere else, their journey is extended to include this location after leaving the GP surgery and before going home; we call this extended travel. The travel burden is then defined as the difference between the extended travel and the base travel. In Fig 1 we show a diagram of how these distances are defined.

The actual computations of distance were made at street level using OpenStreetMap data [11] and the Open Source Routing Machine (OSRM) software [12]. All the distances we compute were the shortest possible walking routes, as computed by OSRM. Note that, while the 2011 census data reports that 74% of the households in England and Wales have access to at least one car [13], using walking distances provides a base measure that represents all patients. Furthermore, since we only model extra distance, this is a suitable measure also for patients that live relatively far from their closest GP (e.g. in rural areas) and might travel to their GPs by public transport (e.g. bus or taxi) but would still have to walk to a testing facility.

### Location-allocation model

To find the optimal location of the testing facilities and the allocations of patients to facilities, we made use of a facility location optimisation model. Facility location is a mature field in Operations Research [14] devoted to solving problems which involve deciding where to place certain resource providers to cover the demand for a certain area. There exist many different variants and extensions of such models, we refer the reader to [15,16] for two literature reviews of facility location in a health care context.

Our proposed method is an integer programming model that calculates the number of PoC machines that should be available at each GP surgery or pharmacy location (including no machines at certain locations). The model allocates patients to one of these locations in order to minimise the total overall travel, and ensuring that patients never have to travel more than a predefined maximum distance, that we denote by T.

Both objectives are considered in a lexicographic order, *i*.*e*. the priority is to determine the minimum number of machines necessary and, once this is determined, the assignments of patients to testing locations are decided in order to minimise their travel burden, weighted by population.

A detailed mathematical formulation of the model is given in **S1 Appendix**. The model was implemented in Python and solved with Gurobi 8.0.0, see [17] for the relevant scripts.

### Model configurations

In this section we describe the different configurations under which we solved the optimisation model. We identified four configuration categories: geographical location, candidate locations for placing the tests, maximum travel burden and testing demand. They are summarised in Table 1.

**Table 1:** Summary of the factors evaluated in the different experiments of the model.

Category	Levels
Region	Urban, Rural
Max. travel burden (T)	1, 500, 1000, 2000 (m)
Testing demand	High, Low
Candidate locations	GP surgeries, Pharmacies, Both

For contrast, we considered two urban locations (Southampton and Oxford) and two rural locations (Isle of Wight and Lincolnshire). These are based on the boundaries of different UK administrative regions (eg. Unitary Authorities or Counties). In Table 2 we list some of their key statistics, while in **S1 Appendix** we describe how these regions were defined in more detail.

**Table 2:** Key statistics of the considered geographical regions.

Geographical Region	Population	Area (km^2^)	CCGs	Number of OAs
Southampton	254275	50.2	1	766
Oxford	170350	57.5	1	463
Isle of Wight	139798	382.7	1	466
Lincolnshire	1073343	6963.3	6	3422

The base case of placing one machine at each GP surgery is represented by T = 1 m (we allowed 1 m to avoid possible errors by the route calculation software). This setting was solved only for GP-based facilities, as patients referred to a pharmacy would always incur in some extra travel. Note that, even for larger values of T, imposing a maximum allowed travel burden when we consider only pharmacies could still result in an infeasible model, particularly in rural regions. This would happen if, for some patients, going to the nearest pharmacy is still a burden of a distance larger than T. In these cases, to avoid infeasibility, we removed this constraint for these patients and assigned them to the nearest pharmacy. The percentage of patients that are allocated within the limit set by T is reported in our results.

### Patient and public involvement

The National Institute for Health Research (NIHR) Community Healthcare MedTech and In Vitro Diagnostics Collaborative (MIC) Antimicrobial Resistance (AMR) patient and public involvement group has been actively engaged in discussions with a member of the research team while the study was being developed. Their views and concerns about the features of importance to patients regarding access to CRP PoC tests have been taken into account in our modelling approach, and they have also advised on the dissemination approach resulting in modifications to improve the clarity of our results and conclusions.

## Results

The following tables show the results of the optimisation model for all the combinations of the described configurations over the two urban (Table 3) and two rural (Table 4) areas.

**Table 3:** Model results for urban areas. PS is the percentage of patients that can be served with the proposed maximum travel burden, T is the maximum travel burden allowed, Mach is the number of required testing machines, ATB is the average travel burden for the patients (m) and MU (%) is the highest capacity utilisation observed across all opened facilities, measured as the expected tests over the testing capacity of that location.

				Low testing demand			High testing demand	
Candidate sites	PS	*T*	*Mach*	*ATB*	*MU*	*Mach*	*ATB*	*MU*
**Southampton**								
GP	100	1	42	0	2.0	42	0	41.1
	100	500	38	18	2.0	38	18	41.1
	100	1000	32	70	2.0	32	70	41.1
	100	2000	18	456	3.1	18	456	64.5
Pharmacy	65.2	500	33	464	2.0	33	464	41.2
	84.4	1000	31	477	2.4	31	477	49.1
	99.6	2000	17	721	2.9	17	721	60.8
Both	100	500	38	18	2.0	38	18	41.1
	100	1000	31	113	2.4	31	113	49.1
	100	2000	16	551	3.2	16	551	66.4
**Oxford**								
GP	100	1	24	0	2.1	24	0	44.3
	100	500	24	0	2.1	24	0	44.3
	100	1000	21	63	2.2	21	63	45.8
	100	2000	17	267	3.0	17	267	62.8
Pharmacy	72.7	500	23	399	2.1	23	399	44.3
	91.4	1000	18	465	2.1	18	465	44.3
	94.3	2000	15	497	2.3	15	497	47.4
Both	100	500	24	0	2.1	24	0	44.3
	100	1000	19	179	2.2	19	179	45.8
	100	2000	17	257	3.0	17	257	62.8

**Table 4:** Model results for urban areas. PS is the percentage of patients that can be served with the proposed maximum travel burden, T is the maximum travel burden allowed, Mach is the number of required testing machines, ATB is the average travel burden for the patients (m) and MU (%) is the highest capacity utilisation observed across all opened facilities, measured as the expected tests over the testing capacity of that location.

				Low testing demand			High testing demand	
Candidate sites	PS	*T*	*Mach*	*ATB*	*MU*	*Mach*	*ATB*	*MU*
**Isle Of Wight**								
GP	100	1	26	0	1.6	26	0	33.9
	100	500	24	8	1.6	24	8	33.9
	100	1000	22	65	1.9	22	65	38.8
	100	2000	18	316	3.1	18	316	65.4
Pharmacy	74.9	500	20	709	1.9	20	709	40.0
	85	1000	20	706	1.9	20	706	40.0
	90	2000	17	840	3.4	17	840	70.2
Both	100	500	24	8	1.6	24	8	33.9
	100	1000	21	124	2.2	21	124	45.9
	100	2000	18	316	3.1	18	316	65.4
**Lincolnshire**								
GP	100	1	228	0	2.4	228	0	50.4
	100	500	213	8	2.4	213	8	50.4
	100	1000	193	38	2.7	193	38	56.6
	100	2000	160	245	5.5	161	213	76.8
Pharmacy	55.4	500	132	3025	2.8	132	3025	58.1
	68.7	1000	118	3044	3.1	118	3044	65.6
	79.4	2000	103	3093	3.1	103	3093	65.6
Both	100	500	211	13	2.4	211	13	50.4
	100	1000	188	65	2.7	188	65	56.6
	100	2000	156	276	5.5	157	243	76.8

### Differences across geographical locations

**Fig 2:**
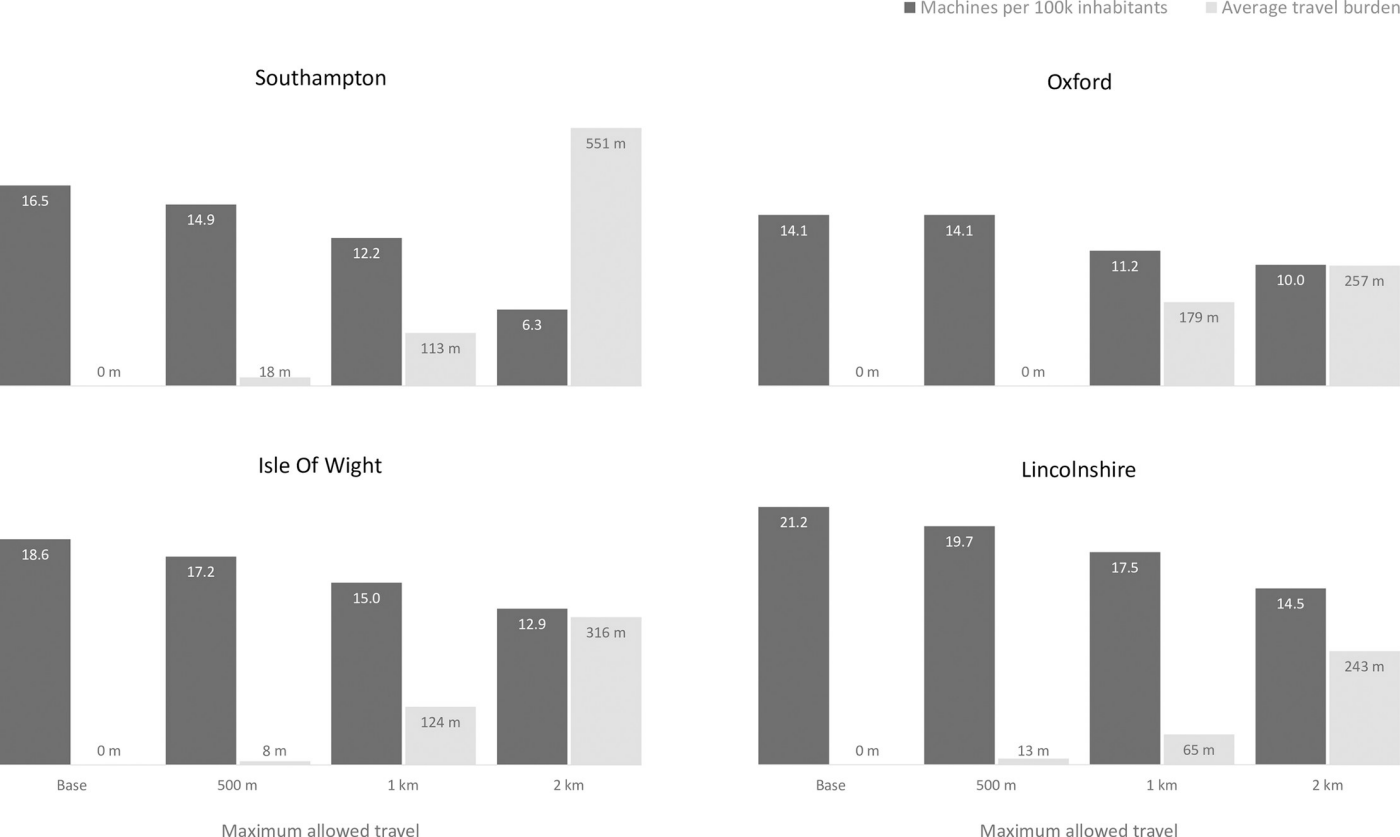
Comparison of the requirement of machines per 100k inhabitants and average travel burden for different locations and limits on travel. Results based on a high testing demand estimate and with both GP surgeries and pharmacies as candidate locations.

Fig 2 illustrates the required number of machines per 100,000 inhabitants and the average travel burden for patients, for a scenario with high testing demand and using both GP surgeries and pharmacies as candidate locations. For the same patient travel burden, rural areas need more testing facilities per person than urban areas. Average travel burden tends to be significantly lower than the travel limits, as there would be only a limited number of patients are assigned to testing facilities significantly far from their usual GP surgeries.

### Effect of testing demand

In general, varying the testing demand estimate from low to high had negligible effect on the number of machines required. In the Southampton, Isle of Wight and Oxford examples the number of machines required was identical. In the low testing demand scenario, the machines had low utilisation (the maximum utilisation was under 6%). The high testing demand scenario resulted in a substantially higher utilisation of machines. The utilisation remained under 80% in all regions.

### Effect of distance limit

Moving from assigning every patient to their closest GP, to allowing them to travel 500 m to other GPs, led to reductions in the number of machines required of up to 15 machines (Southampton 4, Oxford 0, Lincolnshire 15, Isle of Wight 2).

The largest savings were observed when both pharmacies and GP surgeries were potential facilities, combined with a maximum travel distance of 2000 m (with low testing demand the machine reductions compared to the base case were: Southampton 26, Oxford 7, Lincolnshire 72, Isle of Wight 8).

**Fig 3:**
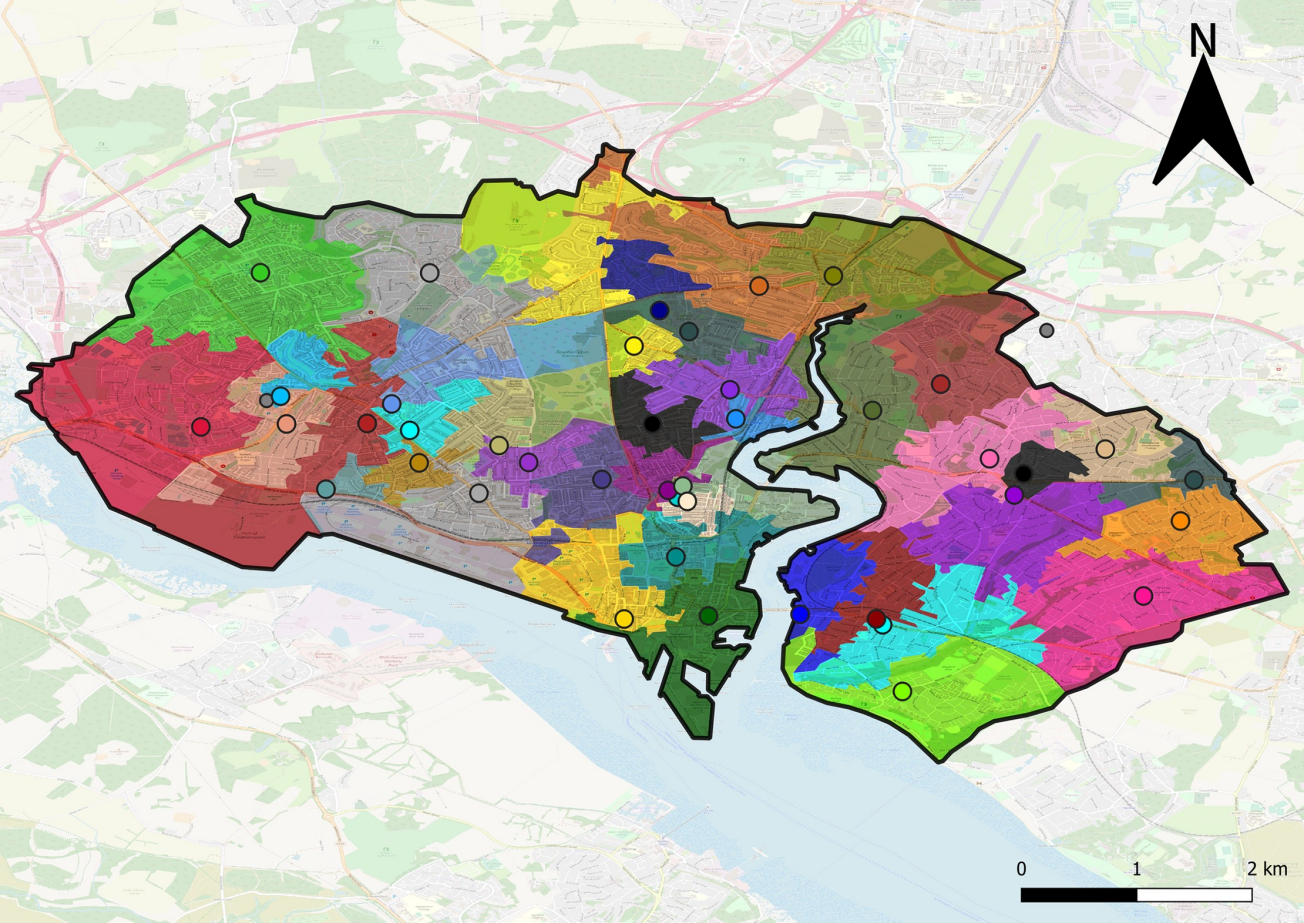
Assignment of patients to GP surgeries for the city of Southampton. Considering a high testing demand estimation and T = 1 m. Each circle represents a GP surgery, same colour indicates assignments. Smaller grey circles indicate that no machine was allocated to that GP surgery. This assignment requires 42 machines in total. Map data copyrighted OpenStreetMap contributors and available from https://www.openstreetmap.org.

**Fig 4:**
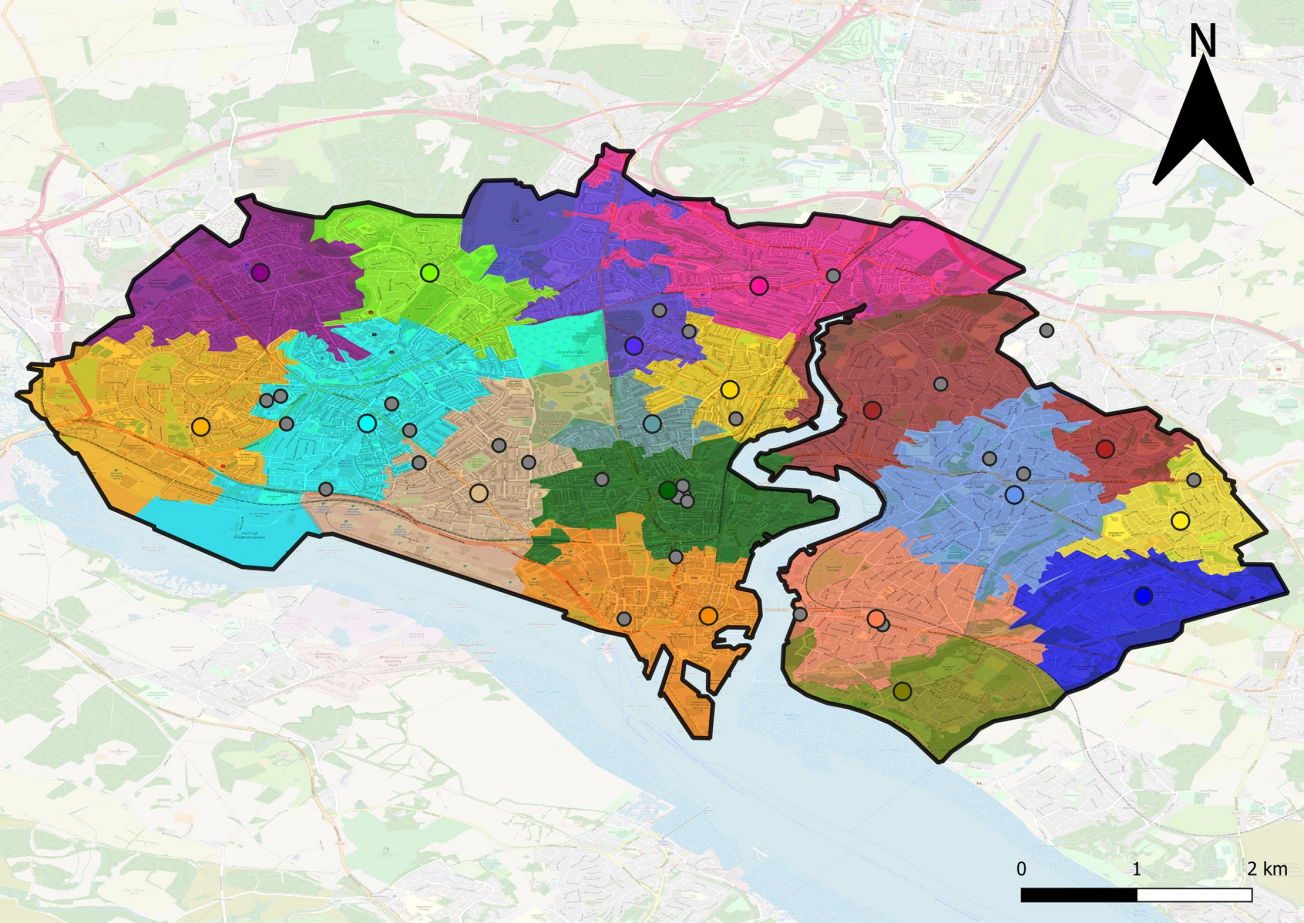
Assignment of patients to GP surgeries for the city of Southampton. Considering a high testing demand estimation and T = 2000. Each circle represents a GP surgery, same colour indicates assignments. Smaller grey circles indicate that no machine was allocated to that GP surgery. This assignment requires 18 machines in total. Map data copyrighted OpenStreetMap contributors and available from https://www.openstreetmap.org.

The actual assignment effect of increasing the maximum travel distance allowed is illustrated by Fig 3 (T = 1 m) and Fig 4 (T = 2 km) for the city of Southampton, where only GP surgeries are considered.

Note that, even in the case where all patients are allocated to be tested at their closest GP, some surgeries do not have any machines assigned (as seen in Fig 3). This can happen if, for every population weighted centroid of the output areas, there is another closer surgery.

### Effect of allowing different candidate facilities

A pharmacy only model was unable to serve all patients under the travel restrictions for any of the locations tested. A number of output areas have a large travel burden to access their nearest pharmacy. This is especially relevant for rural areas (Isle of Wight 12.9 km, Lincolnshire 39.9 km), but also true in urban areas (Southampton 2.2 km, Oxford 3.7 km).

## Discussion

### Summary

We propose a mathematical facility location-allocation model which can be used by commissioners to plan implementation layouts minimising investment and travel burden for patients. Our results suggest that it is possible to achieve great reductions in the number of PoC machines required to deliver this testing approach across a CCG with little travel impact for patients. The model shows that for all regions there is a trade-off between increasing travel requirements for patients and reducing the costs associated with testing machines. Commissioners can use this model as a decision support system to guide the investment needed to roll out a CRP PoC testing network in an area, while ensuring the overall travel burden is minimised.

### Strengths and limitations

This study advances the literature on CRP PoC testing by considering the geographical challenges and the advantages of its widespread implementation. Our results add to previous studies that have focused on the efficiency of CRP testing and on the barriers and facilitators of its adoption. It provides a theoretical ground for establishing service delivery models based on operating with shared resources over a geographic region. Furthermore, it provides a practical tool which can be used in any area to reproduce these analyses and inform practice. To facilitate further studies and reuse we have published all of our code online [17].

**Fig 5:**
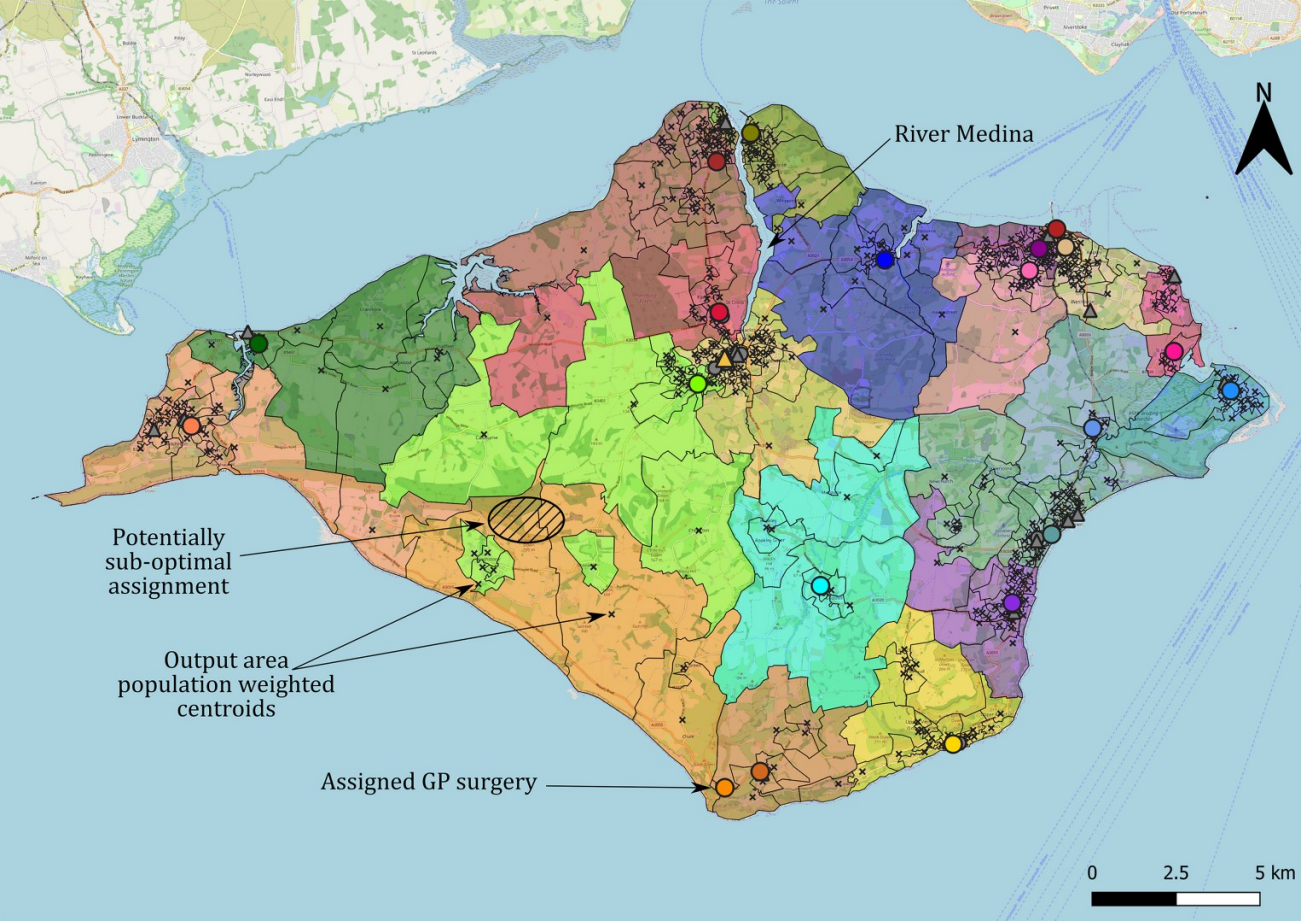
Resulting assignment for the Isle of Wight, illustrating some potentially sub-optimal assignments. This instance was solved for T = 1000, low testing demand and with facilities allowed to be located at GP surgeries (represented by circles, coloured if used, grey if not) and pharmacies (represented by triangles, coloured if used, grey if not). Output areas are delimited by black lines, with their population weighted centroid marked with an “x”. They are coloured matching the colour of the PoC facility their patients have been assigned to. Map data copyrighted OpenStreetMap contributors and available from https://www.openstreetmap.org.

There are some limitations in the way we modelled the displacement of patients. First, we assumed as a starting point the population weighted centroids of the OAs as provided by the census data. In dense urban areas these are likely to be close to the inhabitants of that area, but in rural areas there might be a significant deviation. A clear example of this is Fig 5, where some OAs were allocated in a counter-intuitive manner, as this was a sensible assignment for their population weighted centroids, but possibly inadequate for some patients of the OA living far from the population weighted centroid.

We assumed that patients would go to their closest GP, but this is not necessarily always the case, as some people might choose to be registered somewhere else (e.g. close to their workplace). Furthermore, our definition of travel burden does not take into account patients potentially travelling to pharmacies as part of their routine and, therefore, pharmacy assignments are always subject to travel burden (as opposed to some GP assignments). This means that we have modelled the worst case scenario, where patients might travel to a pharmacy for a test and get a negative result, meaning their journey did not result in the need to collect a prescription.

Nevertheless, our model considers actual walking distances and therefore takes into account geographical features. This can be appreciated in Fig 5, where patients living in the north part of the Isle of Wight near the Medina River were allocated across the river (e.g. the olive green area) because there is a bridge nearby, but this assignment did not happen far from the bridge (blue and red areas).

Finally, our model is concerned with geographical location only, and we do not take into account the implications and costs related to recruitment of extra staff, training, disruption of usual workflow in GP surgeries and pharmacies, etc., that would certainly be relevant if a CRP testing network is deployed.

### Comparison with existing literature

While we are not aware of similar location studies for CRP PoC tests, location problems are common in health care, for example a location of sexual health clinics [18] or the placement of defibrillators for cardiac arrest [19]. The latter study considers a robust model (taking into account the uncertainty surrounding the location of cardiac arrests). We chose not to include uncertainty as, while there is uncertainty in the demand for CRP tests, our results show that testing capacity, even in our conservative estimates, is not a constraint driving the results of the optimisation, as opposed to geographical location. Furthermore, the flexible nature of deploying CRP PoC testing (as opposed, for example, to building a new hospital) makes it possible to run the model every time more data is available and reconfigure the system accordingly (by transferring, acquiring or decommissioning equipment).

### Implications for research and practice

Past research [6,7] has identified cost and clinician time as barriers against the widespread implementation of CRP PoC. An efficient location of tests over a geographical area together with a system where patients are referred to a testing location with delayed prescriptions may alleviate the time pressure on GPs performing tests, as well as reduce the initial investment on equipment and ongoing costs of machine maintenance for commissioners.

Commissioners might make use of this model to determine the budget for rolling out POC CRP across a certain geographical area and to decide where to deploy the testing machines, for a maximum travel burden which is considered adequate, ensuring both minimum costs and a fair distribution of facilities. Furthermore, the results indicate that implementation models led solely by pharmacies might greatly limit the access of some patients to CRP testing. Especially in rural areas, the nearest pharmacy might imply a large detour for patients to be tested.

One possible avenue for future research is cost evaluation. While there is some work in the cost [20] and cost-effectiveness of CRP PoC testing [21], we are not aware of an analysis that takes into account different implementation models for entire geographic areas testing network. Our results might provide more information on the cost-effectiveness of CRP testing over a wider area.

In England, our models are highly relevant to the recently announced NHS primary care networks [22]. These are groups of general practices based in areas with populations ranging from 30,000 to 50,000. Their focus is to enhance the collaboration between health care services in the area and, therefore, the network model could provide the infrastructure needed to share medical records, booking systems, administrative costs and testing results where POC machines are placed in a limited number of practices. Planning tools such as ours could support the distribution of limited funds for point of care testing in order to ensure fair and equitable access for patients and their families.

More widely, the model might be applicable in any region where population estimates are available with sufficiently small granularity (such as in the case of output areas). It could also consider other type of facilities that might be suitable for POC testing (hospitals, for example), or other types of POC tests.

## Supporting information

S1 Appendix Online appendix. Also available at [17].
(PDF)
