# Supplementary material for: A mathematical model for designing networks of C-Reactive Protein point of care testing
Source: PLoS One. 2019 Sep 17;14(9):e0222676. doi: 10.1371/journal.pone.0222676 (PMC6748430; doi:10.1371/journal.pone.0222676)
Supplement: S1 Appendix — Also available at [17]. (PDF) [file pone.0222676.s001.pdf]

A Estimation of testing capacity

CRP tests are intended to be used in primary care. There are a number of different products available, but all typically provide a reading of the CRP concentration in a small blood sample in less than 5 minutes [1, 2]. A required input for the model is the number of tests a single machine could perform per week. This figure is highly dependent on the setting where it would be located. The daily capacity for testing will be affected by the distribution of arrival times for patients, the availability and skill of assigned staff, as well as maintenance tasks such as cleaning the equipment or fetching new cartridges. Since this information is unknown, in order to provide a sensible estimate, we based our calculation on the average number of consultations GPs perform per day. We use as a reference the British Medical Association (BMA), which considers that 35 should be the maximum number of consultations that a GP does per day [3] (although a recent Pulse survey found the actual figure to be 41.5 consultations per day on average [4]). For a five-day week, this leads to the estimate of a capacity of 175 tests per machine per week. The testing is usually shorter than a GP consultation and therefore this figure is likely to underestimate the real capacity.

B Estimation of CRP testing demand

In order to calculate the demand for tests in each geographical area, we used details of the population as well as the incidence of lower respiratory tract infection (LRTI) and historical testing demand data. Population estimates are available from the Office for National Statistics (ONS), in particular we use the mid-year estimates for 2016 [5]. As a spatial reference, we use 2011 Census Output Areas (OAs) and their population weighted centroids. Population weighted centroids are a point that summarises the location of the population within an output area, more information on how they are obtained can be accessed in [6]. We estimated the demand for tests using two different sources.

The first source is the NICE clinical guideline *Respiratory tract infections (self-limiting): prescribing antibiotics* [7], that states that it is estimated that one quarter of the population visits the GP with a respiratory tract infection each year. For a 52 week year, this leads to a rate of

$$\alpha_1 = \frac{1}{4 \cdot 52} = 0.0048$$

tests per person and per week. This figure is likely to overestimate the real incidence, as the diagnosis of some patients would be clear without need for testing. Furthermore, certain groups of patients could be excluded from the target population (for example, those suffering from inflammatory diseases such as Chronic obstructive pulmonary disease (COPD), who would always have a high CRP reading). Thus, we use α_1 as an upper bound of the real estimate.

The second source is the study [8], that describes a trial of CRP PoC testing in five intervention GP surgeries where CRP tests were available to GPs. The

total number of patients registered in these practices was 63743. During the course of three winter months (November 2016 to January 2017, 12 weeks), they recorded the usage of 176 tests.

Using this data, we have calculate the total number of tests per person and per week by:

$$\alpha_2 = \frac{176}{12 \cdot 63743} = 0.000230091 \tag{1}$$

This estimation is much smaller than the previous and, possibly, too conservative if we compare it to hypothetical future setting where CRP PoC are widely available and all health care professionals are aware of and trained on their use. For this reason, we use it as a lower bound of the real quantity and solve the optimisation model with both values, to see how implementation challenges depend on testing demand.

C Support information on geographical data sources

C.1 Geographical regions

The actual geographical areas used in our study are listed in Table 1.

Table 1: Geographical areas and key statistics. Populations are based on the mid 2016 ONS estimates.

Area	Administrative region	Population	Output areas
Southampton	Unitary authority	254275	766
Oxford	City	170350	463
Isle of Wight	Unitary authority	139798	466
Lincolnshire	Ceremonial county	1073343	3422

C.2 Adapting datasets to their relevant regions

The datasets for GP surgeries and branches [9] contain addresses, but not GPS coordinates. In order to obtain these, we first reduced them to the areas we consider (Southampton, Oxford, Lincolnshire and the Isle of Wight). This was done by considering only some CCG regions, as shown in

Table 2.

From the reduced list, we have only included those entries marked as "Active", to avoid using closed or proposed surgeries.

The actual location of each surgery or branch was then obtained by using the Google Maps API and using the online tool [10].

The exception is the following entry, which has been manually coded as the automatic geocoding was suspected innacurate:

Address: '`YULE-SMITH AL - 003, GLENTHAM METHODIST CHAPEL, CAENBY ROAD, GLENTHAM, LINCOLNSHIRE, LN8 2EQ, UK`'
Location (WGS 1984): '`53.397700, -0.494957`'

Table 2: Clinical Commisioning Groups (CCGs) used to identify the GP surgeries in each geographical area.

**Name**	**Code**	**Area**
NHS Southampton CCG	10X	Southampton
NHS Oxfordshire CCG	10Q	Oxford
NHS Isle of Wight CCG	10L	Isle of Wight
NHS Lincolnshire East CCG	03T	Lincolnshire
NHS Lincolnshire West CCG	04D	Lincolnshire
NHS South Lincolnshire CCG	99D	Lincolnshire
NHS South West Lincolnshire CCG	04Q	Lincolnshire
NHS North East Lincolnshire CCG	03H	Lincolnshire
NHS North Lincolnshire CCG	03K	Lincolnshire

To avoid duplicate entries in our final datasets for GP surgery locations, we have used the the integrate feature of the ArcGIS software package to merge points that were within a distance of $25m$ from each other. Note that this integration only affects at the base case, where the number of machines is greatly reduced by removing too similar locations.

D Optimisation model

The problem we are modelling involves two key decisions: how many facilities to open at each candidate site and to which facility should each patients from each output area be assigned to. These type of models, where both the number and location of facilities, as well as the assignment are optimised are called fixed charge location models (see [11] for a recent review of their methodology).

Each of these two decisions is reflected in a set of decision variables. For each potential facility location, denoted by the index $j \in \{1, ..., F\}$, we define an integer variable y_j that represent the number of facilities opened at location j. In a similar fashion, for each output area $i \in \{1, ..., D\}$ and each location $j \in \{1, ..., F\}$ we define a binary variable x_{ij} that takes the value 1 if the patients from location i are tested at facility j and 0 otherwise. If we denote as B_{ij} the travel burden of patients from output area i referred to a facility located at j, and as d_i the demand for tests in an output area i, the optimisation model is as follows:

$$minimise \quad z = \bar{B}\sum_{j=1}^{F} y_j + \sum_{j=1}^{F}\sum_{i=1}^{D} B_{ij} d_i x_{ij} \tag{2}$$

Subject to:

$$\sum_{i=1}^{D} x_{ij} d_i \leq y_j C \qquad j = 1, \ldots, F \tag{3}$$

$$\sum_{j=1}^{F} x_{ij} = 1 \qquad i = 1, \ldots, D \tag{4}$$

$$x_{ij} \in \{0, 1\} \tag{5}$$

$$y_j \in \mathbb{N} \tag{6}$$

Where C is the testing capacity of a single machine. The objective function, equation (2), considers two different objectives in lexicographic order. First it will find out what is the minimum number of machines needed and, among all of the possible allocations with that number, it will choose the one that minimises the overall travel. The number of machines is an integer number obtained by $\sum_{j=1}^{F} y_j$. The overall travel burden can be calculated as $\sum_{j=1}^{F}\sum_{i=1}^{D} B_{ij} d_i x_{ij}$. In a standard fixed charge model, both quantities are related to their monetary cost, an the model minimises overall cost. In our case, we assign a cost of $\bar{B}$ units to the first part. This quantity is an upper boundary on the total possible travel burden, which ensures that the optimal solution prioritises minimising the total number of machines over the travel considerations. The value of $\bar{B}$ can be calculated based on the estimates or demand in each location and their maximum allowed travel selected for the model configuration.

Finally, d_i are the estimated tests needed for output area i. These are calculated with the value of estimated population in the Output Area times the demand estimate being used (α_1 or α_2). Note that this is a expected value of tests per week and it might not necessarily be an integer.

The restrictions on maximum allowed travel burden (T) can be imposed by adding the following constraints to the model:

$$x_{ij} = 0, \forall i, j \textit{ such that } d_{ij} > T \tag{7}$$

Note that imposing (7) effectively removes some decision variables from the model.

References

[1] National Institute for Health and Care Excellence (NICE). Alere Afinion CRP for C-reactive protein testing in primary care; 2016. Available from: https://www.nice.org.uk/advice/mib81.

[2] National Institute for Health and Care Excellence (NICE). QuikRead go for C-reactive protein testing in primary care; 2016. Available from: https://www.nice.org.uk/advice/mib78.

[3] British Medical Association. Controlling workload in general practice; 2018. Available from: https://www.bma.org.uk/advice/employment/gp-practices/service-provision/controlling-workload-in-general-practice.

[4] Julia Gregory. GPs have almost twice the safe number of patient contacts a day; 2018. Available from: http://www.pulsetoday.co.uk/home/finance-and-practice-life-news/gps-have-almost-twice-the-safe-number-of-patient-contacts-a-day/20035863.article.

[5] Office for National Statistics. Census Output Area population estimates; 2017.

[6] Office for National Statistics. Population Weighted Centroids Guidance; 2017. Available from: http://geoportal1-ons.opendata.arcgis.com/datasets/b20460edf2f3459fa7d2771eacab51fc.

[7] National Institute for Health and Care Excellence (NICE). Respiratory tract infections (self-limiting): prescribing antibiotics; 2008. Available from: https://www.nice.org.uk/guidance/CG69.

[8] Johnson M, Cross L, Sandison N, Stevenson J, Monks T, Moore M. Funding and policy incentives to encourage implementation of point-of-care C-reactive protein testing for lower respiratory tract infection in NHS primary care: a mixed-methods evaluation. BMJ open. 2018 10;8(10):e024558. Available from: http://www.ncbi.nlm.nih.gov/pubmed/30366918http://bmjopen.bmj.com/lookup/doi/10.1136/bmjopen-2018-024558%0Ahttp://www.ncbi.nlm.nih.gov/pubmed/30366918.

[9] NHS Digital. GP and GP practice related data; 2018. Available from: https://digital.nhs.uk/services/organisation-data-service/data-downloads/gp-and-gp-practice-related-data.

[10] GPS Visualizer's Easy Batch Geocoder: Convert addresses to coordinates;. Available from: http://www.gpsvisualizer.com/geocoder/.

[11] Fernández E, Landete M. Fixed-Charge Facility Location Problems. In: Location Science. Cham: Springer International Publishing; 2015. p. 47–77. Available from: http://link.springer.com/10.1007/978-3-319-13111-5_3http://dx.doi.org/10.1007/978-3-319-13111-5_3.
